# AlphaFold-Guided Semi-Rational Engineering of an (*R*)-Amine Transaminase for Green Synthesis of Chiral Amines

**DOI:** 10.3390/biom15101435

**Published:** 2025-10-10

**Authors:** Xiaole Yang, Xia Tian, Ruizhou Tang, Jiahuan Li, Xuning Zhang, Tingting Li

**Affiliations:** 1 Jiangsu Key Laboratory of Marine Pharmaceutical Compound Screening, Jiangsu Ocean University, Lianyungang 222005, China; 2023221014@jou.edu.cn (X.Y.); 2022221072@jou.edu.cn (X.T.); 2023221009@jou.edu.cn (R.T.); 2022221039@jou.edu.cn (J.L.); 2 Jiangsu BestEnzymes Biotech Co., Ltd., Lianyungang 222005, China; xuningzhang6@gmail.com; 3 Jiangsu Institute of Marine Resources Development, Lianyungang 222005, China

**Keywords:** amine transaminase, AI-assisted protein engineering, biocatalysis, chiral amines

## Abstract

Chiral amines are vital structural motifs in pharmaceuticals and agrochemicals, where enantiomeric purity governs bioactivity and environmental behavior. We identified a novel (*R*)-selective amine transaminase (MwoAT) from *Mycobacterium* sp. via genome mining, which exhibits activity toward the synthesis of the chiral amine (*R*)-1-methyl-3-phenylpropylamine. The enzyme displayed optimal activity at pH 7.0 and 40 °C, with high thermostability and solvent tolerance. Using an AlphaFold3-guided semi-rational engineering strategy integrating molecular docking, alanine scanning, and saturation mutagenesis, residue L175 was pinpointed as critical for substrate binding. The resulting L175G variant exhibited a 2.1-fold increase in catalytic efficiency (k_cat_/K_m_) and improved thermal stability. Applied to the asymmetric synthesis of (*R*)-1-methyl-3-phenylpropylamine—a precursor for the antihypertensive drug dilevalol and potential scaffold for crop protection agents—the mutant achieved 26.4% conversion with ≥99.9% ee. The enzyme also accepted several ketones relevant to agrochemical synthesis, underscoring its versatility. This work delivers an engineered biocatalyst for sustainable chiral amine production and demonstrates an AI-assisted protein engineering framework applicable to both medicinal and agricultural chemistry.

## 1. Introduction

Chiral amines are vital building blocks in numerous pharmaceuticals and agrochemicals, where stereochemical configuration critically determines their bioactivity, selectivity, and environmental fate [1,2]. In pharmaceuticals, (*R*)-1-methyl-3-phenylpropylamine serves as a key intermediate for the synthesis of the antihypertensive drug dilevalol [3], and in agricultural chemistry, structurally related chiral amines are incorporated into herbicides, insecticides, and fungicides, where enantiomeric purity can strongly influence target specificity, toxicity, and environmental degradability [4,5,6].

Conventional approaches for the synthesis of chiral amines typically rely on asymmetric chemical catalysis or kinetic resolution techniques. While these methods are practically useful, they often suffer from inherent limitations, including a low atom economy, harsh reaction conditions, and dependence on expensive noble metal catalysts [7,8,9]. In contrast, enzymatic catalysis is emerging as a green and sustainable strategy for producing optically pure amines due to its mild conditions, high stereospecificity, and environmental compatibility [10,11,12].

Among various biocatalysts, transaminases have demonstrated remarkable potential in industrial applications due to their broad substrate scope and independence from external cofactor regeneration [13,14]. These enzymes utilize pyridoxal 5′-phosphate (PLP) as a coenzyme and operate via a “ping-pong” mechanism to catalyze the transfer of an amino group from an amine donor to a ketone acceptor, thereby enabling asymmetric amination reactions [15,16,17]. In particular, (*R*)-selective amine transaminases (*R*-ATAs) enable the production of enantiomerically pure chiral amines with high stereospecificity, offering unique advantages in the synthesis of optically active pharmaceutical intermediates [18,19,20]. However, the number of naturally occurring *R*-ATAs remains limited, and most display poor substrate adaptability, moderate thermal stability, and limited tolerance to organic solvents, restricting their industrial scalability [21,22,23,24].

To address these limitations, recent advances in protein engineering and computational biology have provided new strategies for *R*-ATA discovery and optimization. Strategies such as rational design and directed evolution have proven effective in enhancing enzyme catalytic activity, broadening substrate scope, and improving stability [25,26,27,28]. In particular, the integration of AI-based structure prediction tools, such as AlphaFold [29], with molecular docking and molecular dynamics simulations, allows the identification of key residues for substrate binding and catalysis, providing directional guidance for enzyme engineering [30,31]. Concurrently, bioinformatics approaches based on sequence homology alignment and conserved domain analysis have emerged as powerful methods for efficiently mining novel transaminases [32,33]. For example, Höhne et al. developed a domain-focused predictive model that successfully identified *R*-ATA candidates from thousands of protein sequences, providing an effective platform for new enzyme discovery [32].

In the present study, we identified a novel *R*-ATA from *Mycobacterium* sp., expanding the limited *R*-ATA repertoire. Furthermore, we demonstrate the semi-rational engineering and application of this enzyme for the asymmetric synthesis of (*R*)-1-methyl-3-phenylpropylamine, a substrate with practical relevance for pharmaceuticals and agrochemicals. Compared with previous reports employing whole-cell biotransformations [34], we directly utilized the purified enzyme, allowing detailed characterization and optimization.

## 2. Materials and Methods

### 2.1. Materials

All chemical reagents used in this study were of analytical grade or higher. The MwoAT gene was codon-optimized according to the preferences of Mycobacterium and synthetically constructed. It was subsequently subcloned into the pET-15b (+) expression vector. The recombinant plasmid was transformed into *Escherichia coli* BL21(DE3) competent cells for protein expression. The transaminase substrate 4-phenyl-2-butanone and the amine donor (*R*)-2-aminoheptane were both purchased from Aladdin Biochemical Technology Co., Ltd. (Shanghai, China).

### 2.2. Gene Mining and Cloning

Using the amino acid sequence of the *R*-ATA from *Arthrobacter* sp. KNK 168 [35] as a template, we performed BLASTP searches against the NCBI database to identify homologous sequences [36]. Applying an 80% sequence identity threshold yielded a cluster of 97 non-redundant sequences. Multiple sequence alignment was conducted using MUSCLE, followed by phylogenetic tree construction via the neighbor-joining method in MEGA 12.0, to assess the evolutionary relationships between these proteins and other type IV ATAs. Comparative alignment with the *R*-ATA from *Arthrobacter* sp. KNK 168 (PDB: 3WWH) revealed conserved amino acid motifs characteristic of the MwoAT enzyme. The MwoAT target gene was synthesized by General Biosystems (Chuzhou, China), cloned into the pET-15b (+) expression vector, and transformed into *E. coli* BL21(DE3) for recombinant expression.

### 2.3. Protein Expression and Purification

*E. coli* BL21(DE3) cells harboring the recombinant plasmid were cultured in LB medium at 37 °C until the optical density at 600 nm (OD600) reached 0.6. Protein expression was induced by the addition of 0.5 mM IPTG, followed by incubation at 16 °C for 16 h. Cells were harvested by centrifugation and resuspended in lysis buffer (10 mM PBS, pH 7.2), then disrupted by ultrasonication. The recombinant protein was purified using Ni-NTA affinity chromatography and subsequently dialyzed against PBS buffer.

### 2.4. Enzyme Activity Assay and Biochemical Characterization

The standard reaction mixture (500 μL) contained 20 mM (*R*)-2-aminoheptane, 20 mM 4-phenyl-2-butanone, 2 mM pyridoxal 5′-phosphate (PLP), 100 mM triethanolamine buffer (pH 7.15), and transaminase enzyme solution at a final concentration of 10 μg/mL. Reactions were carried out at 40 °C and pH 7.0 for 30 min, and then immediately quenched by boiling at 95 °C in a metal bath for 10 min. After cooling, the samples were centrifuged at 12,000× *g* for 5 min at 4 °C, and the supernatants were collected for enzymatic activity analysis. All assays were performed in triplicate, and results were reported as the mean ± standard deviation.

One unit (U) of enzyme activity was defined as the amount of enzyme required to produce 1 μmol of (*R*)-1-methyl-3-phenylpropylamine per minute at 40 °C and pH 7.0 using 4-phenyl-2-butanone as the ketone acceptor.

To investigate the effect of temperature on enzyme activity, the standard reaction mixture (pH 7.0) was incubated at temperatures ranging from 30 to 65 °C (at 5 °C intervals) for 30 min, followed by immediate inactivation. Residual activity was quantified using HPLC, with the highest activity set as 100%, and the relative activities were plotted accordingly.

To assess thermostability, the enzyme was pre-incubated at 40, 45, 50, 55, and 60 °C for 0, 10, 30, and 90 min at pH 7.0. After treatment, samples were chilled on ice for 2 min and then subjected to the standard reaction at 40 °C. Residual activities were calculated and plotted.

The effect of pH on enzyme activity was determined by replacing the buffer in the standard assay with the following: phosphate buffer (pH 6.0, 7.0), triethanolamine buffer (pH 7.0, 8.0), and glycine–NaOH buffer (pH 8.0, 8.5, 9.0). After standard incubation at 40 °C for 30 min and quenching, residual activity was measured by HPLC, normalized to the maximum observed activity (set as 100%), and plotted.

To evaluate the effect of metal ions, 10 mM final concentrations of NaCl, KCl, MgCl_2_, ZnSO_4_, MnCl_2_, CuCl_2_, CoCl_2_, NiSO_4_, or FeCl_3_ were added to the standard reaction mixture and incubated at 40 °C for 30 min. The reaction without any metal ion was used as a control (relative activity defined as 100%), and the residual activities of each treatment were calculated and plotted.

For organic solvent tolerance assays, 10% (*v*/*v*) of each solvent—ethyl acetate, methanol, acetonitrile, acetone, dimethyl sulfoxide (DMSO), N, N-dimethylformamide (DMF), or tetrahydrofuran (THF)—was added to the standard reaction system and incubated at 40 °C for 30 min. The reaction without solvent served as the control (100% relative activity), and residual activities were calculated and plotted accordingly.

### 2.5. Structural Modeling, Mutant Library Construction, and Functional Analysis

The 3D structure of MwoAT was predicted using AlphaFold3, validated by Rosetta, and assessed with PROCHECK. Semi-flexible molecular docking with AutoDock Vina (PLP as cofactor, 4-phenyl-2-butanone as substrate) identified key residues within 4 Å of the binding pocket, which were further analyzed by alanine scanning and SAR to guide semi-rational engineering.

Eighteen residues were selected for site-directed mutagenesis. Primers were designed with Geneious Prime 2024.0, and mutant plasmids were constructed via seamless cloning (Table S2).

To investigate structural and functional effects, docking of wild-type and mutants was performed using Discovery Studio 2019, analyzing hydrogen bonds, electrostatic interactions, hydrophobic contacts, and unfavorable contacts. Active-site volumes and solvent-accessible surface areas were calculated with CASTp and DoGSiteScorer. MD simulations (30 ns) were conducted in GROMACS 2023 using AMBER99SB-ILDN and TIP3P, with Na^+^/Cl^−^ for neutralization. RMSD and RMSF analyses evaluated structural stability and binding-pocket dynamics [37,38,39,40].

### 2.6. Establishment of HPLC Analytical Method

The enzymatic conversion of 4-phenyl-2-butanone to (*R*)-1-methyl-3-phenylpropylamine catalyzed by MwoAT was quantitatively analyzed using high-performance liquid chromatography (HPLC).

HPLC Conditions for Activity Quantification:

Mobile phase preparation: Phase A consisted of an aqueous solution of 10 mM disodium hydrogen phosphate and 10 mM sodium tetraborate, adjusted to pH 6.1 with dilute hydrochloric acid. Phase B was HPLC-grade acetonitrile.

Instrumentation parameters: A ZORBAX SB-C18 reverse-phase column (4.6 × 150 mm, 5 μm) was used with a column oven temperature of 35 ± 0.5 °C, flow rate of 1.0 mL/min, and UV detection at 260 nm over 15 min.

Elution protocol: Isocratic elution with Phase A and Phase B mixed in a 60:40 ratio. (Figures S1–S3)

Chiral HPLC Conditions for Enantiomeric Excess Determination:

Mobile phase: Phase A, n-hexane (HPLC grade); Phase B, isopropanol (HPLC grade).

Instrumentation parameters: A Chiralpak OD column (250 mm × 4.6 mm, 5 μm) was used with a column temperature of 30 ± 1 °C, a flow rate of 0.5 mL/min, and UV detection at 265 nm for 15 min.

Elution conditions: Isocratic elution with 95% Phase A and 5% Phase B.

Standard Curve Preparation:

Standard solutions were prepared by accurately weighing reference compounds on an analytical balance and diluting to volume in volumetric flasks to obtain a concentration series of 0 mM, 0.2 mM, 0.4 mM, 0.5 mM, 0.6 mM, and 1 mM. Each concentration was prepared in triplicate. All samples were filtered through a 0.22 μm syringe filter before injection. A standard calibration curve was generated using GraphPad Prism, plotting compound concentration (*x*-axis) versus average peak area (*y*-axis) (Figure S4).

### 2.7. Enzyme Kinetics Assay

Kinetic parameters of the transaminase were determined using enzyme solutions at a final concentration of 10 μg/mL. The reaction mixtures contained varying concentrations of the ketone acceptor 4-phenyl-2-butanone (0, 2.5, 5, 10, 20, 40, 70, 100, 150, and 200 mM) and were incubated at 40 °C and pH 7.0 for 30 min. The reactions were terminated by the addition of hydrochloric acid, with 0.5 μL of 4 M HCl added to every 500 μL of reaction mixture. Subsequently, equivalent amount of sodium hydroxide (NaOH) was added to neutralize the solution after the reaction was quenched. The concentration of (*R*)-1-methyl-3-phenylpropylamine in each sample was quantified by HPLC as described above. Relative enzymatic activities at different substrate concentrations were calculated and fitted using a non-linear regression model (Michaelis–Menten equation) in GraphPad Prism 9.0 to generate the kinetic curve.

### 2.8. Thermal Stability Assay

The thermal stability of the transaminase was evaluated using a LightCycler^®^ 480 II real-time PCR system (Roche, San Francisco, CA, USA) with SYPRO Orange-based thermal shift assay. Each reaction contained 5 μg of purified enzyme, 1× SYPRO Orange fluorescent dye, and Buffer D (20 mM triethanolamine, pH 7.5) in a total volume of 50 μL. A negative control was prepared by replacing the enzyme solution with an equal volume of deionized water (dH_2_O). The temperature gradient was programmed to increase linearly from 25 °C to 90 °C, and fluorescence intensity was continuously recorded. The excitation and emission wavelengths were set to 490 nm and 605 nm, respectively.

### 2.9. Substrate Scope Evaluation

To investigate the catalytic potential of the mutant enzyme toward structurally diverse carbonyl compounds and to expand its substrate spectrum, a series of prochiral ketones were selected as alternative acceptor substrates. Each reaction mixture (500 μL) contained 20 mM (*R*)-2-aminoheptane, 20 mM test ketone substrate, 2 mM pyridoxal 5′-phosphate (PLP), 100 mM triethanolamine buffer (pH 7.15), and the mutant transaminase at a final concentration of 10 μg/mL.

The tested ketone acceptors included: (1) 4-phenyl-2-butanone; (2) 4-hydroxy-4-phenyl-2-butanone; (3) 4-methoxy-4-phenyl-2-butanone; (4) 4-(4-hydroxy-3-methoxyphenyl)-2-butanone; (5) 1-acetonaphthone; (6) 4-methylbenzyl acetate; (7) cyclohexanone; (8) 3-acetyl-2-fluoropyridine.

The conversion of each substrate was analyzed using high-performance liquid chromatography (HPLC). Substrate specificity was evaluated by comparing the relative conversion efficiencies against the reference reaction.

## 3. Results

### 3.1. Screening and Characterization of a Novel R-Selective Amine Transaminase

Although *R*-selective amine transaminases (*R*-ATAs) exhibit excellent enantioselectivity, their catalytic activity and thermal stability often fall short of the requirements for industrial-scale applications. To develop an *R*-ATA with both high catalytic efficiency and robust stability, a BLAST search (BLAST 2.16.0) was conducted in the NCBI protein database using a previously reported *R*-ATA from *Arthrobacter* sp. KNK 168 (RCSB PDB ID: 3WWH) as the template. This search identified a putative type IV *R*-ATA from *Mycobacterium* sp. KWX21888.1 (Table S1), hereafter designated as MwoAT. Notably, the function of this type IV *R*-ATA has not yet been reported in the literature.

**Figure 1:**
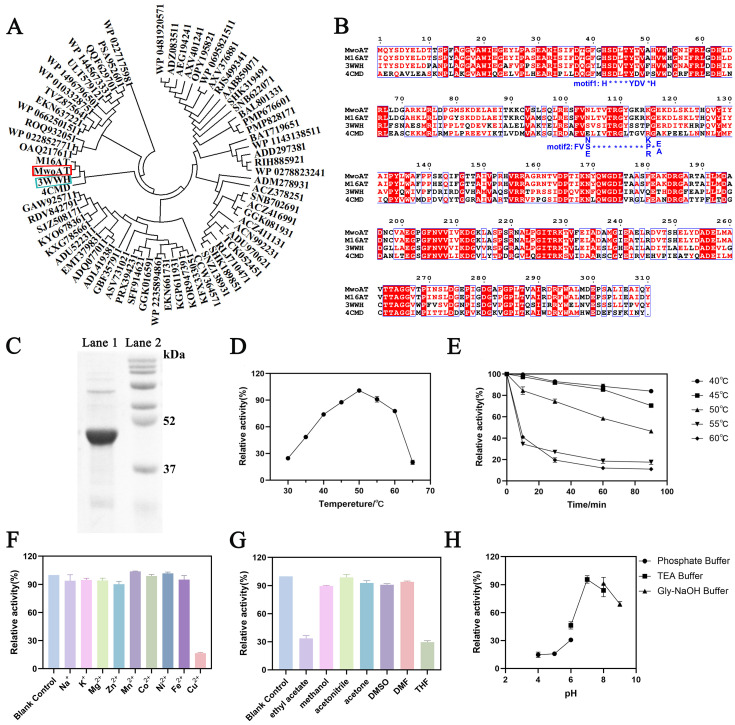
Screening and characterization of a novel *R*-ATA. (**A**) Phylogenetic tree analysis of MwoAT and related transaminases; (**B**) Multiple sequence alignment showing conserved *R*-ATA motifs; (**C**) SDS-PAGE analysis of purified recombinant MwoAT protein (Lane 1: purified transaminase protein; Lane 2: protein molecular weight marker); (**D**) Effect of temperature on MwoAT catalytic activity at pH 7.0 for 30 min; (**E**) Thermostability of enzyme treated at different times and temperatures at pH 7.0 (**F**) Influence of various metal ions on enzymatic activity at 40 °C and pH 7.0 for 30 min; (**G**) Effect of organic solvents (10% *v*/*v*) on enzymatic activity at 40 °C and pH 7.0 for 30 min; (**H**) Effect of pH on enzyme activity at 40 °C for 30 min.

Multiple sequence alignment and phylogenetic analysis (Figure 1A) revealed that MwoAT clusters within the same evolutionary clade as the known *R*-ATA 3WWH, indicating a high degree of sequence similarity. Further analysis of conserved motifs showed that MwoAT harbors characteristic sequence features of *R*-ATAs, including the conserved motif 1: H××××YD[VT]×[STAHP] and motif 2: [FY]V[EQAWNS] [RKFGP]×[STANER] [41] (Figure 1B), suggesting that MwoAT likely possesses *R*-ATA activity.

To verify its expression and catalytic potential, the MwoAT gene was cloned into the pET-15b expression vector containing an N-terminal 6×His tag, resulting in the construction of the recombinant plasmid pET15b-MwoAT. The construct was confirmed by double restriction enzyme digestion and DNA sequencing. Upon expression in *E. coli* BL21(DE3), a soluble protein of approximately 48 kDa—consistent with the theoretical molecular weight—was successfully obtained (Figure 1C), laying the foundation for subsequent functional characterization.

The enzymatic properties of MwoAT were systematically characterized. The enzyme exhibited a temperature-dependent catalytic profile, with maximal activity observed at 50 °C (Figure 1D). Thermal stability assays revealed that approximately 50% of the initial activity was retained after incubation at 50 °C for 90 min, whereas ~85% activity was preserved at 40 °C under the same conditions (Figure 1E). Considering both optimal catalytic activity and thermostability, 40 °C was selected as the optimal reaction temperature for subsequent experiments. Compared with other reported *R*-ATAs, such as M16AT (optimal temperature 40 °C) [42] and 3WWH (optimal temperature 30 °C) [43], MwoAT exhibits relatively high catalytic activity at moderately elevated temperatures, underscoring its potential for industrial applications.

MwoAT displayed optimal activity at pH 7.0, following a typical bell-shaped pH–activity curve (Figure 1H). Enzymatic activity dropped sharply under acidic conditions (pH ≤ 5.0), while ~65% residual activity was maintained at pH 9.0, suggesting that MwoAT remains conformationally stable under neutral to mildly alkaline environments.

In terms of metal ion tolerance, MwoAT showed broad compatibility with most tested metal ions, with the exception of Cu^2+^, which exhibited significant inhibitory effects (Figure 1F). Moreover, the enzyme retained over 90% of its activity in the presence of 10% (*v*/*v*) methanol, acetonitrile, DMSO, acetone, and DMF. However, its activity declined markedly in ethyl acetate and tetrahydrofuran (THF), retaining only 33% and 20% of its original activity, respectively (Figure 1G). This reduction is likely attributed to solvent-induced disruption of the protein’s hydration shell or interference with the structural integrity of the active site.

### 3.2. Catalytic Activity and Thermal Stability Enhancement via Semi-Rational Engineering

Due to the absence of an experimentally determined crystal structure for MwoAT, AlphaFold3 was employed to generate a predictive 3D model. The highest-confidence model was selected for subsequent analysis (Figure S5). Ramachandran plot evaluation indicated that 95.3% of the residues were located in favored regions, and the model achieved an Errat score of 97.015, suggesting high structural reliability suitable for structure-based functional residue identification.

**Figure 2:**
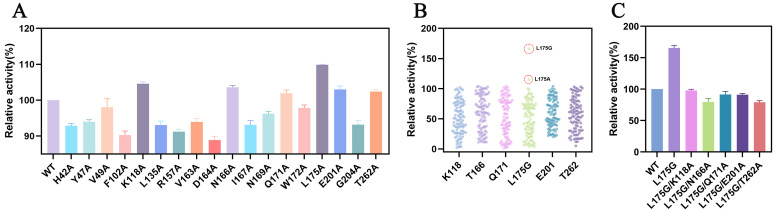
Enhanced catalytic activity of MwoAT through semi-rational engineering. (**A**) Relative activities of alanine-scanning mutants; (**B**) Relative activities of single-point saturation mutants; (**C**) Relative activities of combinatorial double mutants.

To improve the catalytic efficiency of MwoAT, a semi-rational engineering strategy was adopted. Using AutoDock Vina, semi-flexible docking between MwoAT and the substrate 4-phenyl-2-butanone identified 18 residues located within 4 Å of the substrate, including H42, Y47, K118, and L175 (Figure S6). Each of these residues was subjected to alanine scanning mutagenesis, followed by expression, purification, and enzymatic activity evaluation. Six single-point mutants—K118A, T166A, Q171A, L175A, E201A, and T262A—exhibited enhanced catalytic activity compared to the wild-type enzyme. Among them, the L175A variant displayed the most significant improvement (Figure 2A), suggesting that the bulky leucine side chain at position 175 may hinder substrate accommodation due to steric constraints.

Building upon these findings, NNK saturation mutagenesis libraries were constructed for the six beneficial sites. Screening identified the L175G variant as the most active mutant, exhibiting 166% of the catalytic efficiency of the wild-type enzyme (Figure 2B). To explore potential synergistic effects, five double mutants were constructed by combining L175G with each of the other beneficial mutations. However, none of the double mutants surpassed the activity of L175G alone (Figure 2C), indicating possible conformational interference or structural incompatibility between the mutation sites.

To quantitatively evaluate the catalytic performance of MwoAT variants, the kinetic parameters of mutants L175A and L175G toward the substrate 4-phenyl-2-butanone were determined (Table 1). Compared to the wild-type (WT) enzyme, both mutants exhibited enhanced catalytic efficiency (k_cat_/K_m_), with L175A showing a 1.8-fold and L175G a 2.1-fold improvement. Specifically, the Km of L175G decreased from 68.03 ± 1.68 mM (WT) to 42.87 ± 0.43 mM, indicating an increased substrate affinity. Concurrently, the k_cat_ value increased from 236.15 ± 0.21 to 319.20 ± 1.56, reflecting an elevated catalytic turnover rate. These results demonstrate that the L175G mutation enhances both substrate binding and catalytic efficiency.

In addition, differential scanning fluorimetry (DSF) using SYPRO Orange dye was employed to assess the thermal stability of the mutants by determining their melting temperatures (Tm). The WT enzyme exhibited a Tm of 53.8 ± 0.1 °C, whereas L175A and L175G showed significantly elevated Tm values of 57.0 ± 0.1 °C and 56.0 ± 0.1 °C, respectively, suggesting that both mutations confer improved thermodynamic stability to the protein.

### 3.3. Substrate Scope Expansion and Asymmetric Biocatalysis

**Figure 3:**
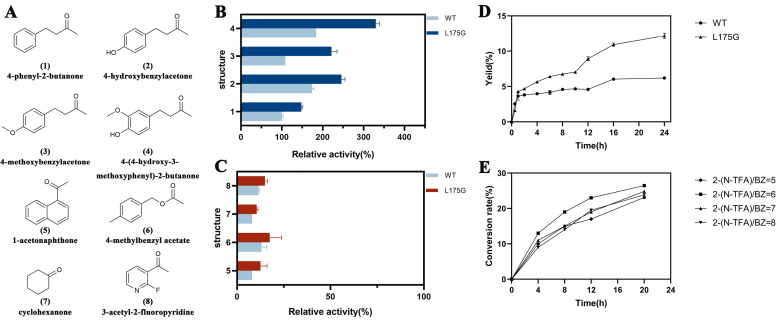
Substrate specificity and stereoselectivity of WT and L175G mutant. (**A**) Structural formulas of different substrates; (**B**,**C**) Comparison of catalytic activities of WT and L175G toward different substrates; (**D**) Time-course product yield comparison between WT and L175G over 24 h; (**E**) Conversion rates at varying amine-to-ketone molar ratios over different reaction times.

To assess the catalytic versatility of MwoAT and its mutant L175G toward structurally diverse ketone substrates, a panel of representative compounds bearing distinct functional groups and steric features was selected for activity screening. As shown in Figure 3B, L175G exhibited enhanced catalytic activity compared to the WT enzyme toward 4-phenyl-2-butanone and its structural analogs. Notably, the mutant also outperformed WT in converting bulky substrates such as 1-acetonaphthone and 3-acetyl-2-fluoropyridine (Figure 3C), indicating that the enlarged active-site pocket in L175G facilitates better accommodation of sterically demanding substrates, thus broadening its substrate specificity.

Further time-course assays comparing WT and L175G revealed that, although the initial reaction trajectories were similar (Figure 3D), the mutant consistently exhibited higher catalytic efficiency. Under identical enzyme loading, the product yield of L175G reached 12.18 ± 0.38% after 24 h, approximately double that of the WT (6.21 ± 0.03%). Additionally, at a fixed ketone concentration of 40 mM, the effect of varying the molar ratio of amine to ketone was investigated. The results demonstrated that a molar ratio of 6:1 (amine: ketone) yielded a maximum conversion rate of 26.4% after 20 h (Figure 3E).

Chiral HPLC analysis confirmed that both WT and L175G maintained strict *R*-stereoselectivity, with enantiomeric excess (ee.) values ≥99.9% (Figures S10–S13). These results indicate a very high optical purity, demonstrating excellent stereoselectivity of the products. The findings also show that the L175G mutation significantly improves catalytic performance without compromising the inherent stereoselectivity, making it a promising biocatalyst for the asymmetric synthesis of chiral amines.

### 3.4. Structural Modeling and Molecular Dynamics Reveal the Mechanism of Enhanced Catalysis

The binding pocket of an enzyme—typically a three-dimensional cavity formed on or within the protein surface—plays a pivotal role in ligand recognition and catalysis. Its geometric parameters (e.g., volume, depth, and aperture) and physicochemical properties (e.g., hydrophobicity, charge distribution) collectively determine substrate specificity and binding affinity [44].

To elucidate the molecular basis of the enhanced catalytic activity and thermostability observed in the L175G mutant, we performed detailed structural modeling and molecular dynamics (MD) simulations. Binding pocket characterization using the DoGSiteScorer module of the ProteinsPlus platform [45] revealed that the pocket volume of L175G increased to 988 Å^3^, compared to 917 Å^3^ in the wild-type enzyme. This expansion of the catalytic cavity likely facilitates better substrate accommodation and alleviates steric hindrance.

**Figure 4:**
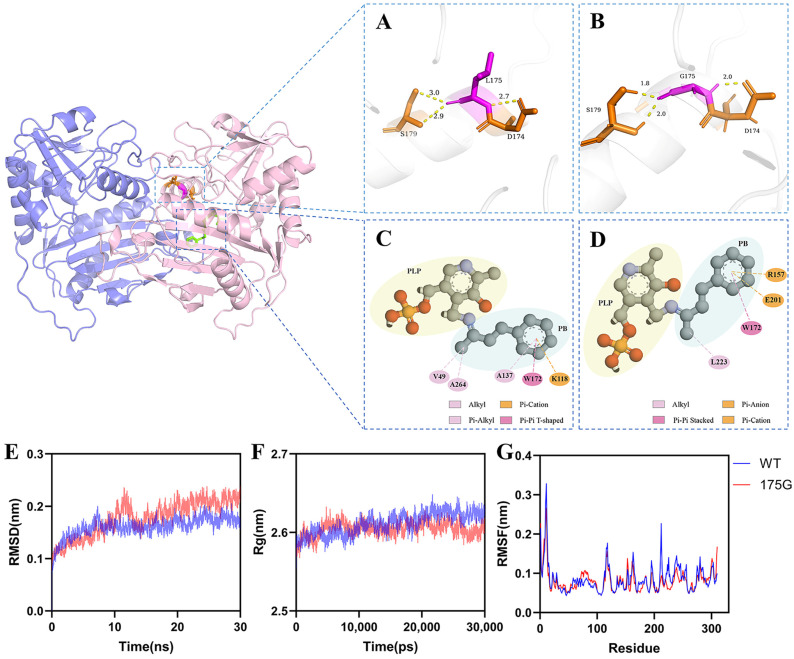
Structural and dynamic analysis of the wild-type and L175G mutant enzymes. (**A**,**B**) Hydrogen bond distance analysis of key active-site residues in the wild-type (**A**) and L175G mutant (**B**) based on modeled protein–substrate complexes; (**C**,**D**) Interaction force analysis of the docked ligand in the active sites of the wild-type (**C**) and L175G mutant (**D**); (**E**) Root-Mean-Square Deviation (RMSD) profiles comparing the structural stability of wild-type and mutant enzymes; (**F**) Radius of gyration (Rg) profiles indicating differences in global compactness; (**G**) Root-Mean-Square Fluctuation (RMSF) analysis reflecting residue-level flexibility; The L175G mutation leads to an enlarged binding pocket, enhanced hydrogen bonding, and increased structural rigidity, contributing to improved catalytic performance and thermostability.

To further probe this mechanistic hypothesis, molecular docking was conducted using the modeled enzyme structure and the PLP–4-phenyl-2-butanone external aldimine intermediate. The substitution of leucine with glycine at position 175 significantly reduced local side-chain bulk, decreasing hydrophobic packing and spatial hindrance at the entrance of the active site. Hydrogen bond analyses showed that the average distances between the substrate and key catalytic residues Asp174 and Ser179 were shortened from 2.7 Å, 2.9 Å, and 3.0 Å in the WT to 2.0 Å, 2.0 Å, and 1.8 Å in L175G, respectively (Figure 4A,B). This optimized hydrogen-bonding network enhances both substrate affinity and the stabilization of transition-state intermediates [46].

Analysis of protein–substrate interactions (Figure 4C,D) revealed that the L175G variant exhibited enhanced interactions with the aromatic ring of the substrate, thereby improving the overall binding stability. The π–π stacking interaction between Trp157 and the substrate’s aromatic ring remained unchanged; however, the π–cation interaction shifted from Lys118 to Arg157. In contrast, the original π–alkyl interaction contributed by Ala137 was replaced by a π–anion interaction involving Glu201. Compared with the π–alkyl interaction, which relies on weak van der Waals forces, the π–anion interaction involves a stronger electrostatic attraction [47,48]. This alteration in the interaction pattern helps stabilize the substrate conformation within the active site, thereby enabling the L175G variant to exhibit higher catalytic activity toward 4-phenyl-2-butanone and its structural analogues.

To elucidate the structural basis for the enhanced thermal stability of the L175G variant, MD simulations were performed on both WT and mutant enzymes. As shown in Figure 4G, the root mean square fluctuation (RMSF) profile of L175G is uniformly lower than that of WT, particularly in the 50–100 and 250–300 residue regions, indicating reduced backbone flexibility and improved structural rigidity. Similarly, the radius of gyration (Rg) of L175G remains consistently smaller throughout the simulation (Figure 4F), suggesting a more compact global conformation, likely due to enhanced hydrophobic packing and a reinforced hydrogen-bond network.

Root-Mean-Square Deviation (RMSD) analysis (Figure 4E) revealed that the L175G mutant reached equilibrium after approximately 10 ns, maintaining lower RMSD values than the wild-type enzyme, thereby corroborating its enhanced structural stability. From a structural perspective, the L175G substitution alleviates steric hindrance imposed by the bulkier leucine side chain, thereby increasing overall rigidity and suppressing conformational unfolding at elevated temperatures [49,50]. These synergistic effects collectively underpin the observed improvement in thermodynamic stability.

## 4. Discussion

In this study, we successfully identified and comprehensively characterized a novel (*R*)-selective amine transaminase (MwoAT) from *Mycobacterium* sp. through genome mining and bioinformatic screening. The enzyme exhibited an optimal pH of 7.0 and a temperature optimum of 40 °C, along with remarkable thermostability and broad tolerance to multiple organic solvents, thereby highlighting its potential for industrial biocatalytic applications.

To overcome the enzyme’s inherent catalytic limitations, we employed an AlphaFold3-guided semi-rational engineering strategy integrating structural modeling, molecular docking, alanine scanning, and saturation mutagenesis, which successfully yielded the L175G variant. This mutant achieved a 2.1-fold improvement in catalytic efficiency (k_cat_/K_m_) over the wild-type enzyme. Structural modeling and molecular dynamics simulations revealed that the L175G substitution relieved steric hindrance, expanded the substrate-binding pocket, and reinforced hydrogen-bond networks involving key catalytic residues (Asp174, Ser179), thus enhancing substrate affinity, turnover rate, and thermal stability.

When benchmarked against prior *R*-ATA engineering efforts, the 2.1-fold improvement achieved here is moderate but meaningful. For instance, Xie et al. reported a 2.8-fold enhancement in AtTA catalytic efficiency through H210N and I77L substitutions combined with disulfide bond formation (M150C and M280C), which also markedly improved thermostability [51]. Similarly, a single-point mutation F113T in an ω-transaminase variant led to a 1.3–1.7-fold increase in catalytic efficiency (k_cat_/K_m_) toward pyruvate and MBA substrates, highlighting that even subtle active-site modifications can significantly modulate enzyme kinetics [52]. Compared with these studies, the L175G variant of MwoAT exhibits a performance level at the mid-to-upper range, with the added benefit of enhanced thermal stability over its wild-type counterpart.

**Scheme 1:**
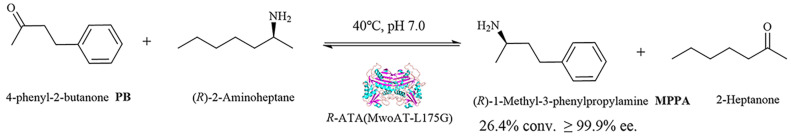
Transamination catalyzed by MwoAT to synthesize (*R*)-1-methyl-3-phenylpropylamine, with yields and enantiomeric excess (ee) of representative reactions indicated.

When applied to the asymmetric synthesis of (*R*)-1-methyl-3-phenylpropylamine—a pharmaceutically and agrochemically important chiral amine intermediate serving as a key precursor to the antihypertensive drug dilevalol and a scaffold for crop-protection agents—the L175G variant achieved a preparative-scale conversion of 26.4% while maintaining ≥ 99.9% enantiomeric excess and strict *R*-stereoselectivity (Scheme 1). Moreover, the substrate scope expansion to aromatic and heteroaryl ketones further underscores the synthetic utility of MwoAT. Importantly, the amine product from substrate 2 serves as an intermediate for Ractopamine synthesis, while the product from substrate 5 is a key precursor for Cinacalcet, demonstrating the pharmaceutical relevance of this biocatalyst.

Although the 4-phenyl-2-butanone is not a natural substrate, MwoAT’s performance demonstrates the feasibility of designing and optimizing *R*-ATAs for non-natural substrates, providing a valuable strategy for expanding the application of these enzymes in industrial biocatalysis. Taken together, these results suggest that MwoAT is not only operationally robust but also an effective biocatalyst for industrially relevant chiral amine synthesis, emphasizing both its novelty and functional advantages.

Despite these promising outcomes, several limitations remain. The relatively low conversion yield (26.4%) may limit immediate industrial applicability. Moreover, the moderate fold-improvement and lack of additive effects in combinatorial mutants suggest mechanistic constraints. We speculate that the wild-type enzyme may already be near its thermodynamic optimum, or that rate-limiting steps involve cofactor binding or product release rather than the targeted residues. The failure of combinatorial mutants likely results from negative epistasis, where beneficial mutations interact unfavorably, creating steric or conformational conflicts that diminish catalytic efficiency. Future efforts could focus on distal allosteric sites or employ mutagenesis strategies designed to minimize such interactions.

To address these challenges, multiple strategies could be explored: (i) use of isopropylamine as an amine donor, enabling continuous acetone removal to shift the reaction equilibrium toward product formation [53]; (ii) implementation of coupled enzymatic systems for in situ by-product removal or cofactor regeneration to improve conversion efficiency [54]; (iii) process intensification via substrate feeding optimization, enzyme immobilization, or reaction medium engineering to enhance yield and operational robustness. Furthermore, the current structural insights rely on AlphaFold3-predicted models rather than experimentally resolved structures, which may limit the precision of active-site geometry and substrate-binding analyses. Future studies incorporating X-ray crystallography, cryo-EM, and iterative combinatorial mutagenesis could further improve enzyme performance while validating the mechanistic basis underlying the observed enhancements.

## 5. Conclusions

Overall, this work delivers an engineered and adaptable biocatalyst for the sustainable production of bioactive chiral amines with applications spanning pharmaceuticals, agrochemicals, and agricultural biotechnology. It also establishes a generalizable “AI-based structure prediction–semi-rational engineering–functional validation–mechanistic interpretation” workflow for enzyme design. This integrated approach provides both theoretical insights and practical tools for developing next-generation biocatalysts that meet the efficiency, selectivity, and environmental sustainability requirements of modern medicinal and agricultural chemistry.

## Figures and Tables

**Table 1:** Kinetic parameters of MwoAT variants toward 4-phenyl-2-butanone. (See Figures S7–S9 for Michaelis–Menten plots).

Enzyme	k_cat_ (min^−1^)	K_m_ (mM)	k_cat_/K_m_ (mM^−1^·min^−1^)
WT	236.15 ± 0.21	68.03 ± 1.68	3.47 ± 0.09
L175A	340.60 ± 3.25	54.25 ± 2.42	6.28 ± 0.34
L175G	319.20 ± 1.56	42.87 ± 0.43	7.44 ± 0.15

## Data Availability

The data that support the findings of this study are available in the Supplementary Materials of this article.

## Supplementary Materials

The following supporting information can be downloaded at https://www.mdpi.com/article/10.3390/biom15101435/s1, Figure S1. Retention time of (R)-1-methyl-3-phenylpropylamine standard in HPLC analysis; Figure S2. Retention time of 4-phenyl-2-butanone standard in HPLC analysis; Figure S3. HPLC chromatogram of the reaction catalyzed by MwoAT; Figure S4. Standard calibration curve for (R)-1-methyl-3-phenylpropylamine; Figure S5. Predicted 3D structure of MwoAT and validation of model quality; Figure S6. Residues within 4 Å of the substrate in the enzyme-substrate complex; Figure S7. Michaelis-Menten curve for wild-type MwoAT; Figure S8. Michaelis-Menten curve for mutant MwoAT-L175A; Figure S9. Michaelis-Menten curve for mutant MwoAT-L175G; Figure S10. Determination of product optical purity; Figure S11. ^1^H NMR spectrum of the catalytic product; Figure S12. ^13^C NMR spectrum of the catalytic product; Figure S13. UPLC-MS analysis of the catalytic product; Table S1. Gene sequence of MwoAT; Table S2. Primer design for site-directed mutagenesis.
