# Supplementary material for: AlphaFold-Guided Semi-Rational Engineering of an (R)-Amine Transaminase for Green Synthesis of Chiral Amines
Source: Biomolecules. 2025 Oct 10;15(10):1435. doi: 10.3390/biom15101435 (PMC12564524; doi:10.3390/biom15101435)
Supplement: Supplementary file 1 [file biomolecules-15-01435-s001.zip › biomolecules-3898638-supplementary.pdf]

Supporting Information

AlphaFold-Guided Semi-Rational Engineering of an (*R*)-Amine Transaminase for Green Synthesis of Chiral Amines

Xiaole Yang[1], Xia Tian[1], Ruizhou Tang[1], Jiahuan Li[1], Xuning Zhang[3], Tingting Li*[1,2]

[1] Jiangsu Key Laboratory of Marine Pharmaceutical Compound Screening, Jiangsu Ocean University, Lianyungang 222005, China; 2023221014@jou.edu.cn (X.Y.); 2022221072@jou.edu.cn (X.T.); 2023221009@jou.edu.cn (R.T.); 2022221039@jou.edu.cn (J.L.)

[2] Jiangsu BestEnzymes Biotech Co., Ltd., Lianyungang 222005, China; xuningzhang6@gmail.com

[3] Jiangsu Institute of Marine Resources Development, Lianyungang 222005, China

* Correspondence: litt@jou.edu.cn

Xiaole Yang and Xia Tian are co-first authors.

Figure

Figure S1. Retention time of (*R*)-1-methyl-3-phenylpropylamine standard in HPLC analysis. ····· 1
Figure S2. Retention time of 4-phenyl-2-butanone standard in HPLC analysis. ······················ 2
Figure S3. HPLC chromatogram of the reaction catalyzed by MwoAT.······························ 3
Figure S4. Standard calibration curve for (*R*)-1-methyl-3-phenylpropylamine.······················ 4
Figure S5. Predicted 3D structure of MwoAT and validation of model quality.······················ 5
Figure S6. Residues within 4 Å of the substrate in the enzyme-substrate complex. ·················· 6
Figure S7. Michaelis-Menten curve for wild-type MwoAT.·· 7
Figure S8. Michaelis-Menten curve for mutant MwoAT-L175A.······································· 8
Figure S9. Michaelis-Menten curve for mutant MwoAT-L175G.······································· 9
Figure S10. Determination of product optical purity.···10
Figure S11. ^{1}H NMR spectrum of the catalytic product. ···11
Figure S12. ^{13}C NMR spectrum of the catalytic product. ··12
Figure S13. UPLC-MS analysis of the catalytic product.···13

Table

Table S1. Gene sequence of MwoAT.··14
Table S2. Primer design for site-directed mutagenesis. ···15

Figure

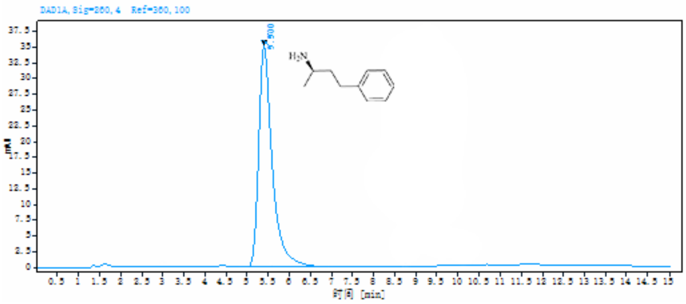

Explanation	DAD1A,Sig=260,4 Ref=360,100		
Retention Time [min]	Peak area	Peak height	Peak height %
5.500	610.47520	33.958	100.00

Figure S1. Retention time of (R)-1-methyl-3-phenylpropylamine standard in HPLC analysis.

The standard of (*R*)-1-methyl-3-phenylpropylamine was analyzed using the HPLC method described in Materials and Methods. A distinct peak was observed at a retention time of 5.500 minutes.

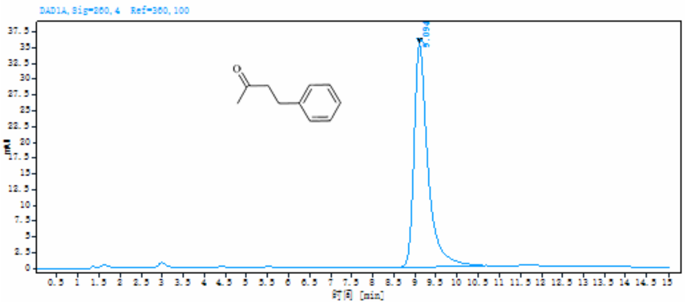

Explanation	DAD1A,Sig=260,4 Ref=360,100		
Retention Time [min]	Peak area	Peak height	Peak height %
9.094	812.89020	35.388	100.00

Figure S2. Retention time of 4-phenyl-2-butanone standard in HPLC analysis.

The 4-phenyl-2-butanone standard was analyzed using the HPLC method described in Materials and Methods, yielding a sharp peak at a retention time of 9.094 minutes.

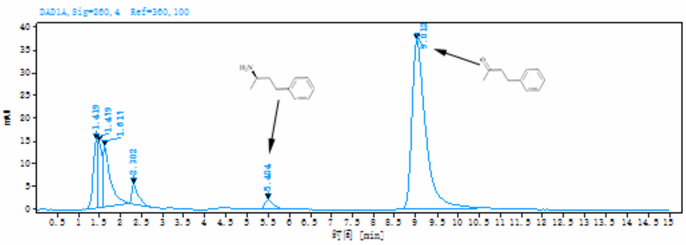

Explanation	DAD1A,Sig=260,4 Ref=360,100		
Retention Time [min]	Peak area	Peak height	Peak height %
5.484	29.13148	2.012	2.34
9.012	848.27975	37.255	43.27

Figure S3. HPLC chromatogram of the reaction catalyzed by MwoAT.

After the enzymatic reaction of 4-phenyl-2-butanone catalyzed by MwoAT, the product (*R*)-1-methyl-3-phenylpropylamine and the substrate were well separated, confirming the reliability and resolution of the HPLC method for product quantification.

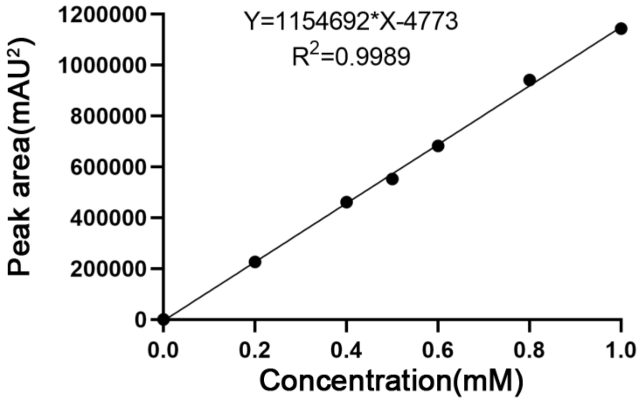

Figure S4. Standard calibration curve for (R)-1-methyl-3-phenylpropylamine.

A standard calibration curve was generated by plotting HPLC peak area against the concentration of (*R*)-1-methyl-3-phenylpropylamine using external standard method. The resulting linear regression equation was: $y = 1154692x - 4773$, with an R^2 value of 0.9989, indicating a strong linear correlation.

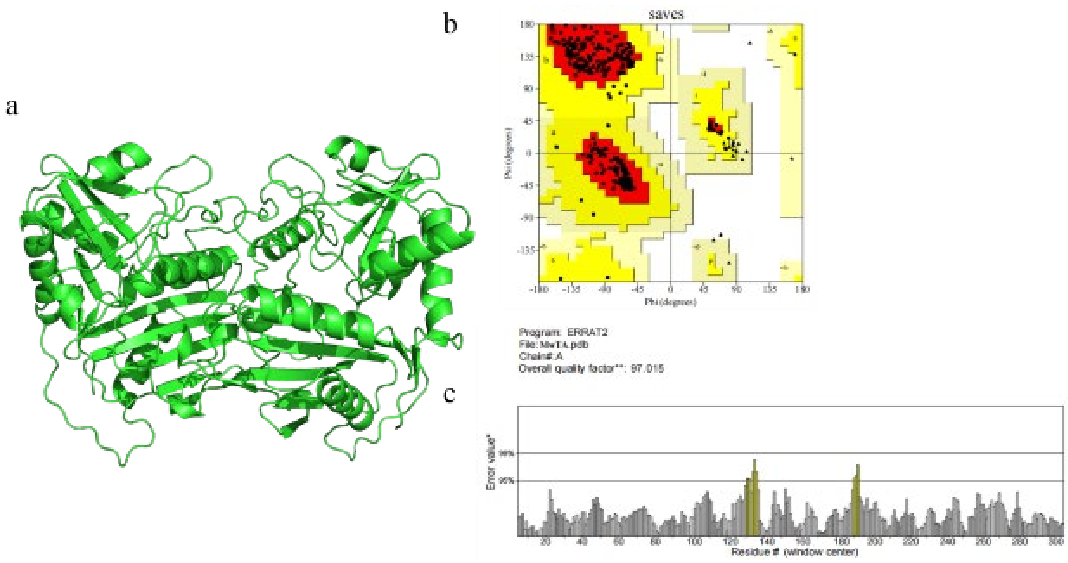

Figure S5. Predicted 3D structure of MwoAT and validation of model quality.

(a) The three-dimensional structure of MwoAT was predicted using AlphaFold3. Among five predicted models, the one with the highest confidence score was selected for further analysis.

(b) Ramachandran plot generated using PROCHECK in the SAVES v6.0 platform showed 95.3% of residues in the most favored region (yellow), 4.7% in allowed regions (light yellow), and 0.0% in disallowed regions (white), meeting the standard for high-quality protein models (>90% in favored regions).

(c) Structural quality evaluation using Errat yielded an overall score of 97.015, significantly higher than the threshold for reliable models (>85%). These results confirm that the predicted MwoAT model is of high quality and suitable for subsequent structural and functional analyses.

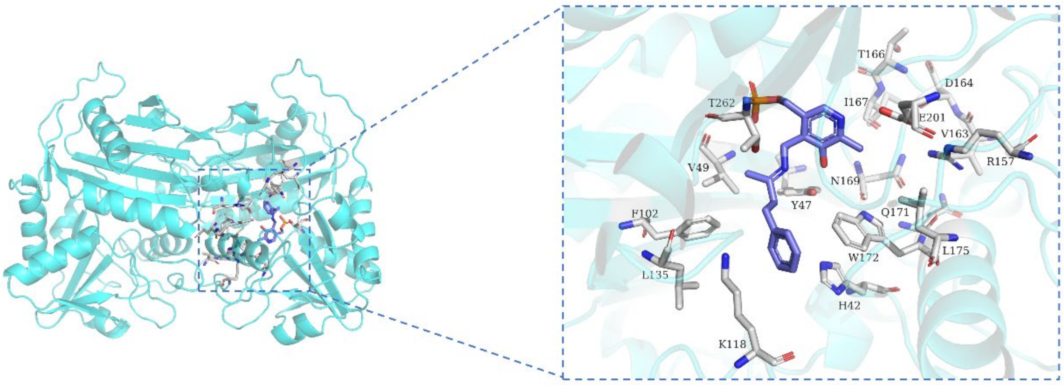

Figure S6. Residues within 4 Å of the substrate in the enzyme-substrate complex.

MwoAT was semi-flexibly docked with 4-phenyl-2-butanone using AutoDock Vina. A total of 18 residues located within 4 Å of the substrate were identified as potential active site residues: H42, Y47, V49, F102, K118, L135, R157, V163, D164, N166, I167, N169, Q171, W172, L175, E201, G204, and T262.

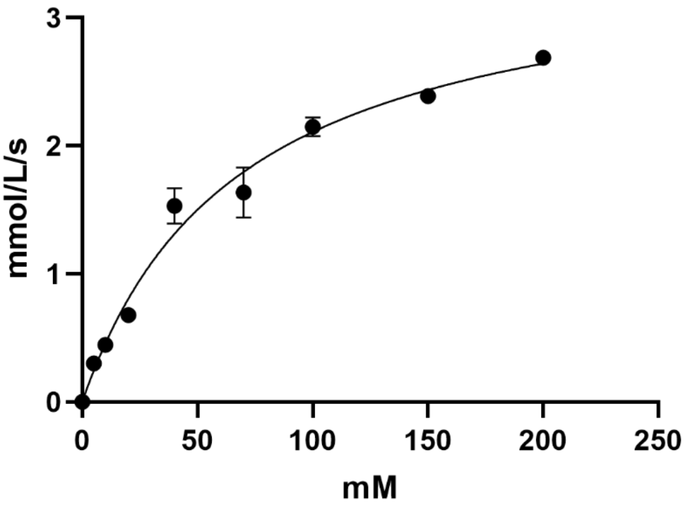

Figure S7. Michaelis-Menten curve for wild-type MwoAT.

The catalytic performance of wild-type MwoAT toward 4-phenyl-2-butanone was analyzed by nonlinear regression fitting to the Michaelis-Menten model. The maximum reaction velocity (V_{max}) was 3.540 mmol·L^{-1}·s^{-1}, and the Michaelis constant (K_m) was 68.03 ± 1.68 mM, with a goodness-of-fit (R^2) of 0.9832, indicating a strong fit between the model and experimental data.

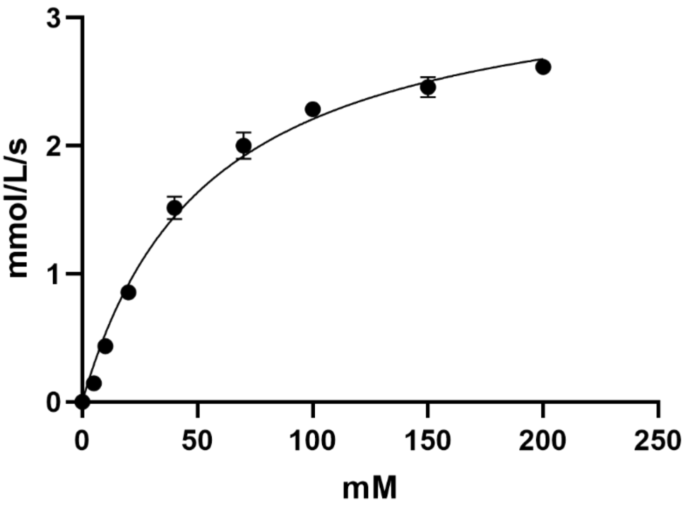

Figure S8. Michaelis-Menten curve for mutant MwoAT-L175A.

The catalytic performance of the MwoAT-L175A mutant was analyzed under identical conditions. The maximum reaction velocity (V_{max}) was 3.405 mmol·L^{-1}·s^{-1}, and the K_m value was 54.25 ± 2.42 mM. The R^2 value of 0.9919 indicates excellent model fitting to the experimental data.

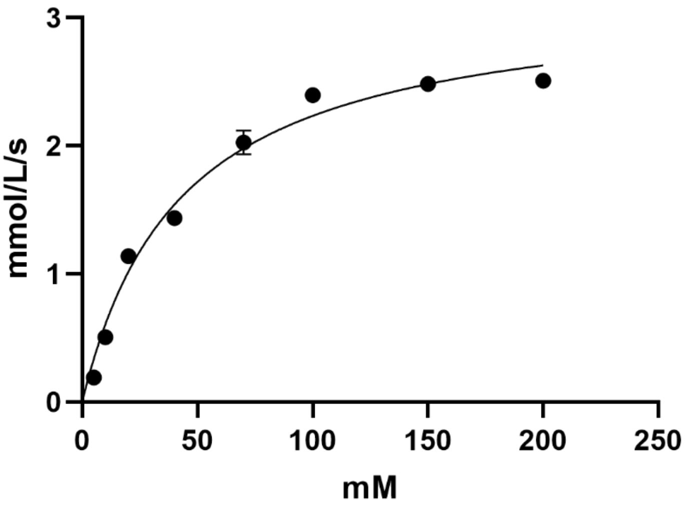

Figure S9. Michaelis-Menten curve for mutant MwoAT-L175G.

The catalytic performance of the MwoAT-L175G mutant was evaluated. The V_{max} was 3.192 mmol·L^{-1}·s^{-1}, and the K_m value was 42.87 ± 0.43 mM. The R^2 value of 0.9821 confirms a good fit to the nonlinear regression model, supporting improved substrate affinity.

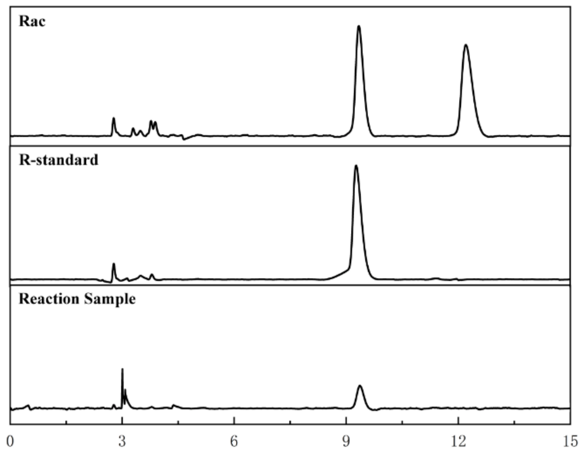

Figure S10. Determination of product optical purity.

Chiral HPLC analysis was performed according to the method described in Materials and Methods. The racemic standard of 1-methyl-3-phenylpropylamine showed two peaks at approximately 9.5 min and 12.5 min. The (*R*)-enantiomer standard eluted at 9.5 min. The purified product from MwoAT-catalyzed conversion of 4-phenyl-2-butanone also showed a single peak at 9.5 min, with no detectable peak at 12.5 min, indicating that the product was (*R*)-1-methyl-3-phenylpropylamine with an enantiomeric excess (ee) ≥ 99.9%.

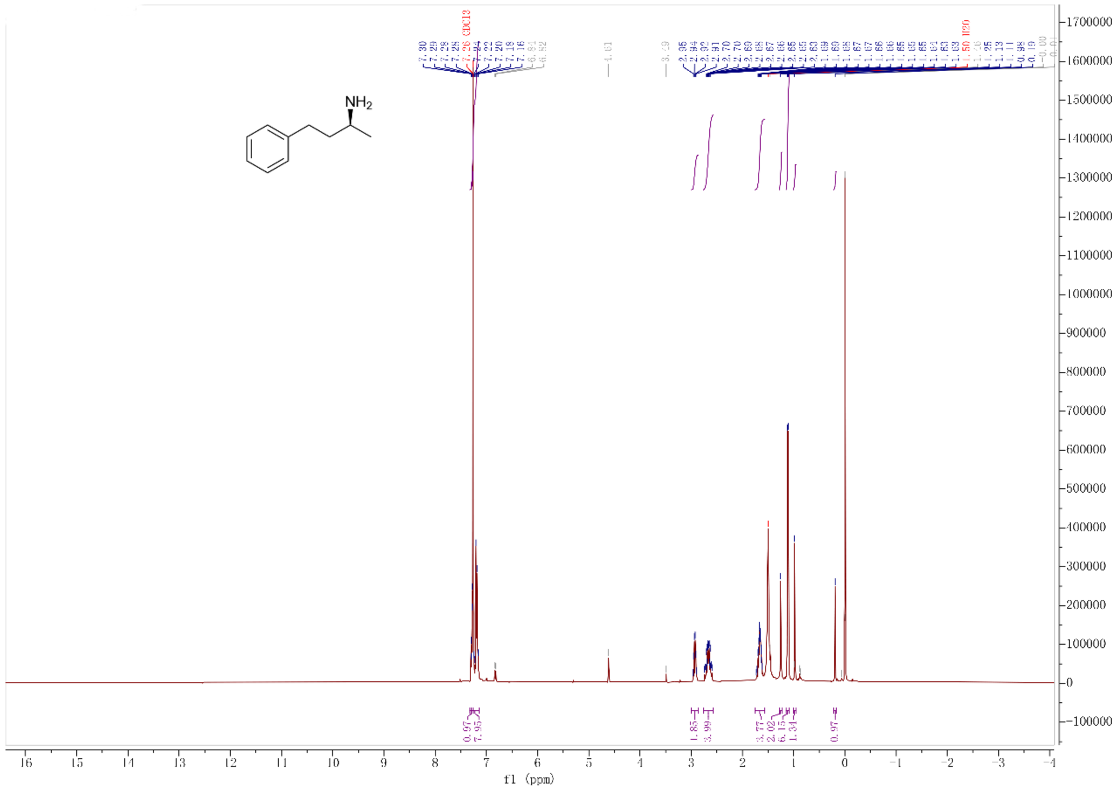

Figure S11. ^{1}H NMR spectrum of the catalytic product.

^{1}H NMR (400 MHz, CDCl3) δ 7.29 (d, J = 6.6 Hz, 1H), 7.30 – 7.14 (m, 8H), 2.93 (h, J = 6.3 Hz, 2H), 2.67 (qdd, J = 13.7, 9.3, 6.7 Hz, 4H), 1.75 – 1.57 (m, 4H), 1.25 (s, 2H), 1.12 (d, J = 6.3 Hz, 6H), 0.98 (s, 1H), 0.19 (s, 1H).

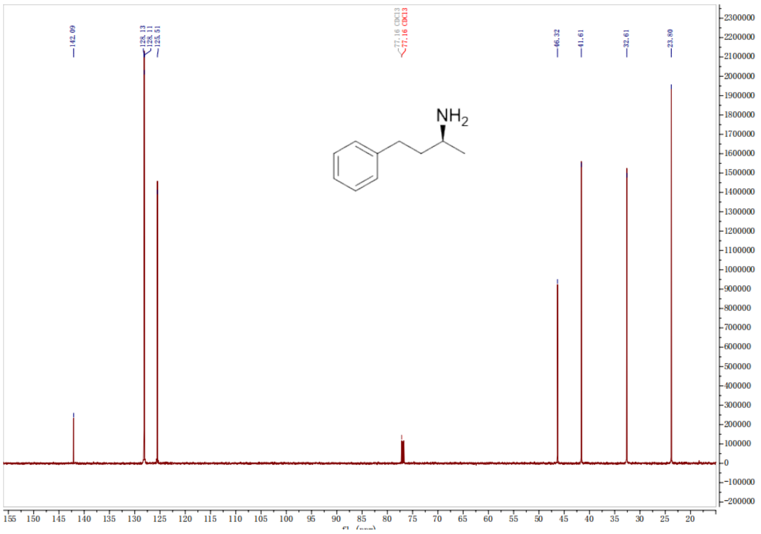

Figure S12. ^{13}C NMR spectrum of the catalytic product.

^{13}C NMR (100 MHz, CDCl3) δ142.09,128.13,128.11,125.51,77.16,46.32,42.61,32.61,23.80.

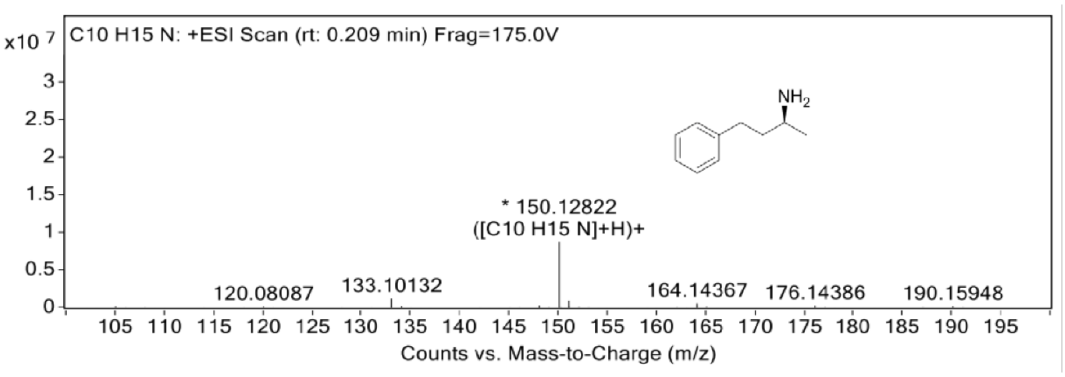

Figure S13. UPLC-MS analysis of the catalytic product.

The UPLC-MS spectrum confirms the expected molecular weight of the product (*R*)-1-methyl-3-phenylpropylamine, consistent with the target structure.

Table

Table S1. Gene sequence of MwoAT.

DNA Sequence	Protein Sequence
ATGCAGTATAGTGATTACGAACTGGATA	MQYSDYELDTTSPFAGGVAWIEGEYLPA
CCACCAGCCCGTTTGCCGGTGGTGTTG	SEAKISIFDTGFGHSDLTYTVAHVWHGNI
CCTGGATTGAAGGTGAATATCTGCCGGC	FRLGDHLDRLLDGARKLRLDPGMSKDE
AAGTGAAGCAAAAATTAGTATTTTTGAC	LAEITKKCVSLSQLRESFVNLTVTRGYGK
ACCGGTTTTGGCCATAGTGATCTGACCT	RKGEKDLSKLTHQVYIYAIPYLWAFPPSE
ATACCGTTGCCCATGTTTGGCATGGCAA	QIFGTTAIVPRHVRRAGRNTVDPTIKNYQ
TATTTTTCGCCTGGGTGACCATCTGGAT	WGDLTAASFEAKDRGARTAILMDADNC
CGTCTGCTGGATGGCGCACGTAAACTG	VAEGPGFNVVIVKDGKLASPSRNALPGIT
CGTCTGGACCCTGGCATGAGCAAAGAT	RKTVFEIADAMGIEAELRDVTSHELYDA
GAACTGGCCGAAATTACCAAAAAATGT	DELMAVTTAGGVTPINSLDGEPIGDGAPG
GTTAGTCTGAGTCAGCTGCGTGAAAGT	PLTVAIRDRFWALMDEPSALIEAIQY
TTTGTGAATCTGACCGTGACCCGTGGCT	
ATGGCAAACGCAAAGGTGAAAAAGATC	
TGAGTAAACTGACCCATCAGGTTTATAT	
CTATGCAATTCCGTATCTGTGGGCATTTC	
CGCCGAGTGAACAGATTTTTGGCACCA	
CCGCAATTGTGCCGCGCCATGTTCGCCG	
CGCCGGTAGAAATACCGTTGATCCGAC	
CATTAAGAATTATCAGTGGGGCGATCTG	
ACCGCAGCAAGCTTTGAAGCAAAAGAT	
CGCGGTGCCCGTACCGCAATTCTGATG	
GATGCCGATAATTGCGTGGCCGAAGGC	
CCGGGTTTTAATGTGGTTATTGTGAAAG	
ATGGTAAACTGGCCAGTCCGAGTCGTA	
ATGCACTGCCGGGCATTACCCGCAAAA	
CCGTGTTTGAAATTGCCGATGCCATGGG	
CATTGAAGCCGAACTGCGCGATGTTAC	
CAGTCATGAACTGTATGATGCCGATGAA	
CTGATGGCAGTGACCACCGCCGGCGGT	
GTTACCCCGATTAATAGCCTGGATGGCG	
AACCGATTGGTGACGGCGCACCGGGTC	
CGCTGACCGTTGCAATTCGTGATCGTTT	
TTGGGCACTGATGGATGAACCGAGTGC	
ACTGATTGAAGCAATTCAGTATTAAGGA	
TCC	

Table S2. Primer design for site-directed mutagenesis.

Primer	Sequence
42A-F	GACACCGGTTTTGGCGCCAGTGATCTGACCTATACC
42A-R	GGTATAGGTCAGATCACTGGCGCCAAAACCGGTGTCAAAA
47A-F	AGTGATCTGACCGCCACCGTTGCCCATGTTTGG
47A-R	CCAAACATGGGCAACGGTGGCGGTCAGATCACTATGGCCA
49A-F	CTGACCTATACCGCCGCCCATGTTTGGCATGGC
49A-R	GCCATGCCAAACATGGGCGGCGGTATAGGTCAGATCACTA
102A-F	CTGCGTGAAAGTGCCGTGAATCTGACCGTGACC
102A-R	GGTCACGGTCAGATTCACGGCACTTTCACGCAGCTGACTC
118A-F	CGCAAAGGTGAAGCCGATCTGAGTAAACTGACC
118A-R	GGTCAGTTTACTCAGATCGGCTTCACCTTTGCGTTTGCCA
135A-F	GCAATTCCGTATGCCTGGGCATTTCCGCCGAGT
135A-R	ACTCGGCGGAAATGCCCAGGCATACGGAATTGCATAGATA
157A-F	CGCCATGTTCGCGCCGCCGGTAGAAATACCGTT
157A-R	AACGGTATTTCTACCGGCGGCGCGAACATGGCGCGGCACA
163A-F	GGTAGAAATACCGCCGATCCGACCATTAAGAAT
163A-R	ATTCTTAATGGTCGGATCGGCGGTATTTCTACCGGCGCGG
164A-F	AGAAATACCGTTGCCCCGACCATTAAGAATTAT
164A-R	ATAATTCTTAATGGTCGGGGCAACGGTATTTCTACCGGCG
165A-F	AATACCGTTGATGCCACCATTAAGAATTATCAG
165A-R	CCACTGATAATTCTTAATGGTGGCATCAACGGTATTTCTACC
